# Case Report: Persistent Primitive Hypoglossal Artery Accompanied by a Basilar Bifurcation Aneurysm Treated by Y-Stent-Assisted Coil Embolization

**DOI:** 10.3389/fneur.2021.621610

**Published:** 2021-03-03

**Authors:** Longhui Zhang, Xiheng Chen, Luqiong Jia, Linggen Dong, Jiejun Wang, Peng Liu, Ming Lv

**Affiliations:** Department of Interventional Neuroradiology, Beijing Neurosurgical Institute, Beijing Tiantan Hospital, Capital Medical University, Beijing, China

**Keywords:** persistent primitive hypoglossal artery, basilar bifurcation aneurysm, wide-neck, Y-stent, endovascular treatment

## Abstract

Successful embolization of a basilar bifurcation aneurysm associated with a persistent primitive hypoglossal artery (PPHA) using Y-stent-assisted coiling.

## Introduction

**Figure 1:**
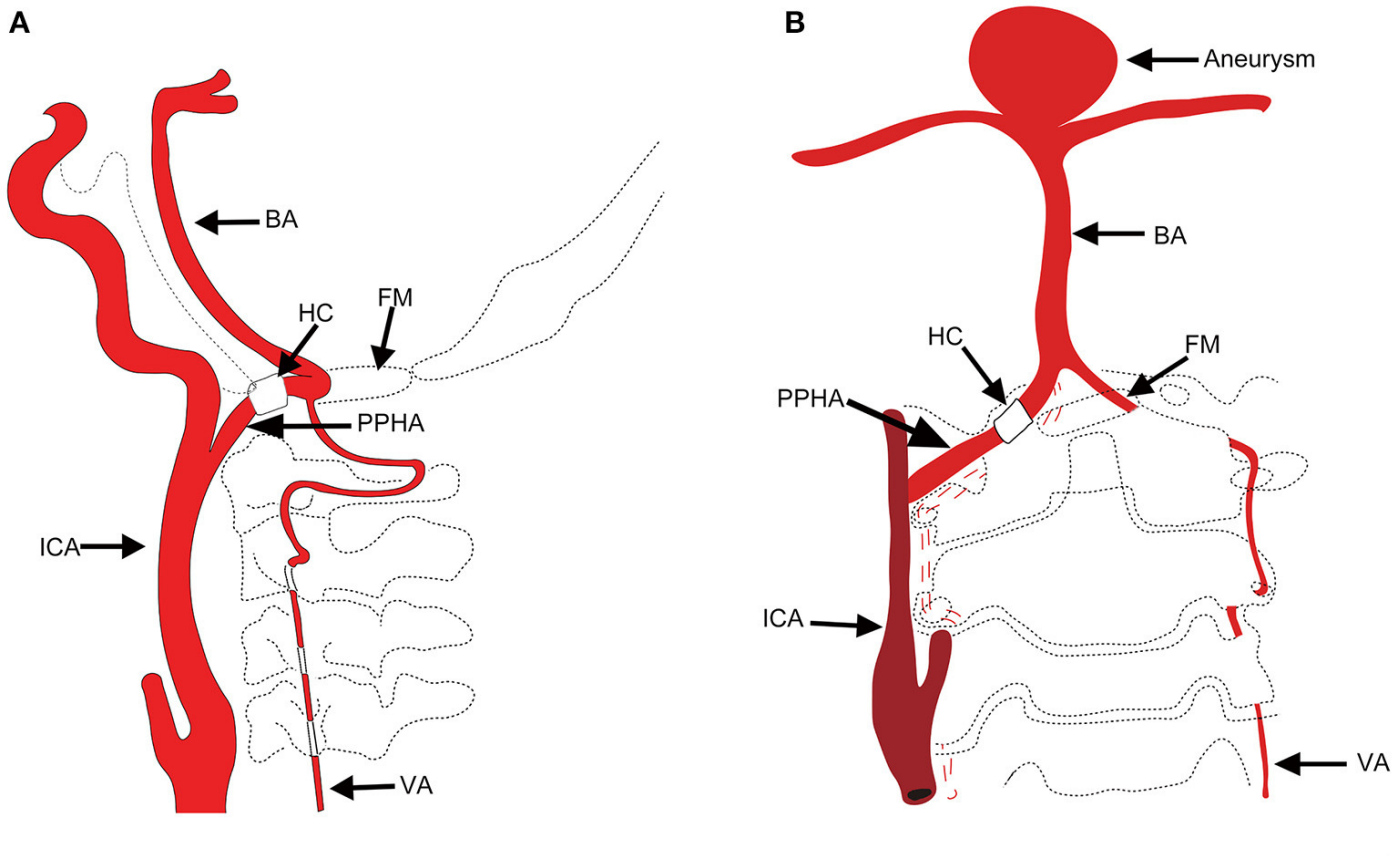
**(A)** Schematic of the PPHA in our patient. BA, basilar artery; HC, hypoglossal canal; FM, foramen magnum; PPHA, persistent primitive hypoglossal artery; ICA, internal carotid artery; VA, vertebral artery. **(B)** The schematic of basilar bifurcation aneurysm.

Carotid–vertebrobasilar anastomoses, known as presegmental arteries in the embryonic period, supply blood from the internal carotid artery (ICA) to the primitive vertebrobasilar system (1). Persistent primitive hypoglossal artery (PPHA) is the second most common primitive communication between the ICA and the basilar artery (BA). The incidence of this persistent carotid–vertebrobasilar anastomosis is 0.027–0.26% (2). Due to an anomalous structure of the vessel wall, posterior circulation blood supply from the ICA, and exposure to unusual hemodynamic stress, PPHA patients often have intracranial anomalies, such as aneurysms (3–9). PPHA disrupts hemodynamic stability between the carotid and vertebrobasilar system. Although the incidence of PPHA accompanying intracranial aneurysms is approximately 26% (10), there are few reports of basilar bifurcation aneurysm. To better characterize PPHA, we created a schematic to summarize this type of carotid–vertebrobasilar anastomosis (Figure 1).

We report a patient with an unruptured wide-neck basilar bifurcation aneurysm accompanied by right PPHA. Although vertebral arteries were present, both were hypoplastic. We advanced a microcatheter via the PPHA, with the help of Y-stent-assisted coiling and successfully treated this aneurysm. Follow-up 6 months after the endovascular treatment using brain digital subtraction angiography (DSA) showed no recanalization of the aneurysm. To our knowledge, the use of Y-stent-assisted coiling to treat PPHA accompanied by basilar bifurcation aneurysm has not been reported previously. Here, we describe the details of the patient's clinical course with a brief review of the relevant literature.

## Case Report

**Figure 2:**
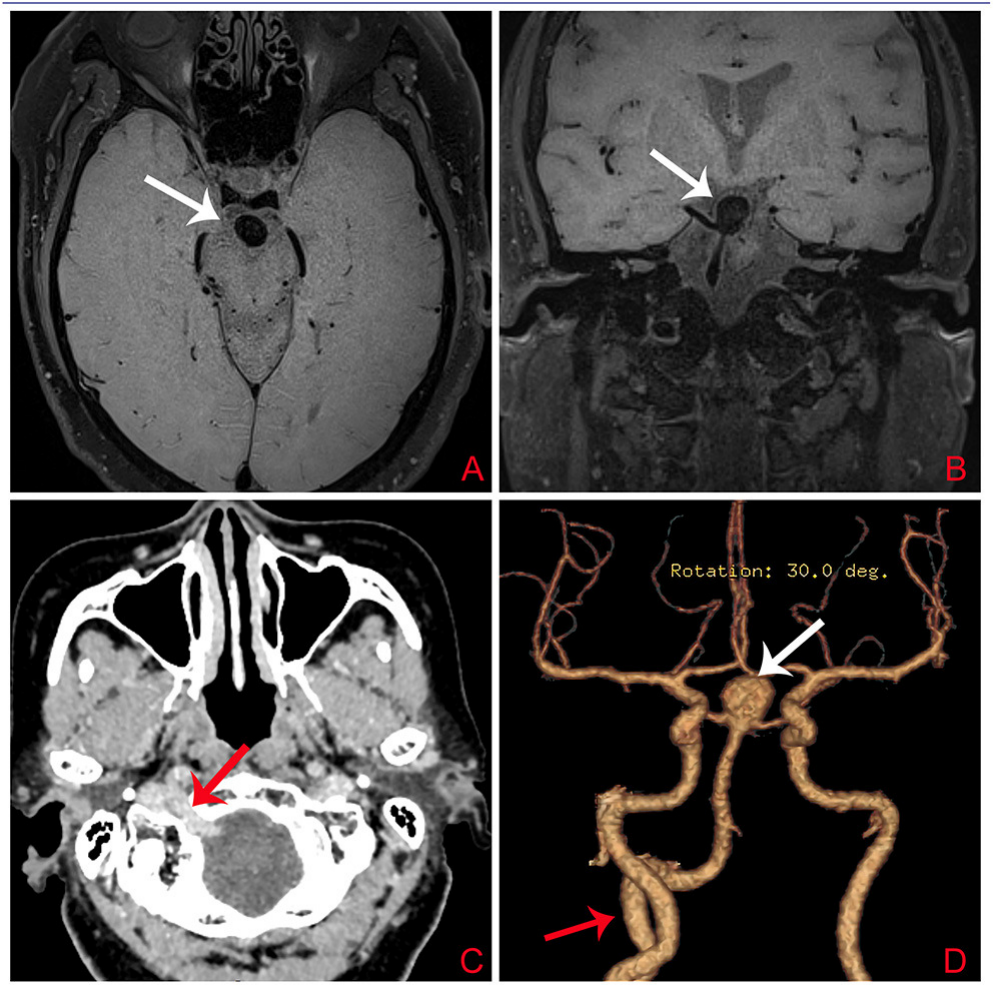
**(A,B)** T1-weighted image (T1WI) showing an aneurysm at the bifurcation of the basilar artery (white arrow). **(C)** Computed tomography angiography demonstrating the PPHA (red arrow) entering the skull after traversing the hypoglossal canal. **(D)** Computed tomography angiography showing the PPHA (red arrow) originating from the right ICA and joining the BA. An aneurysm (white arrow) located at the bifurcation of the basilar artery is visible. PPHA, persistent primitive hypoglossal artery; ICA, internal carotid artery; BA, basilar artery.

**Figure 3:**
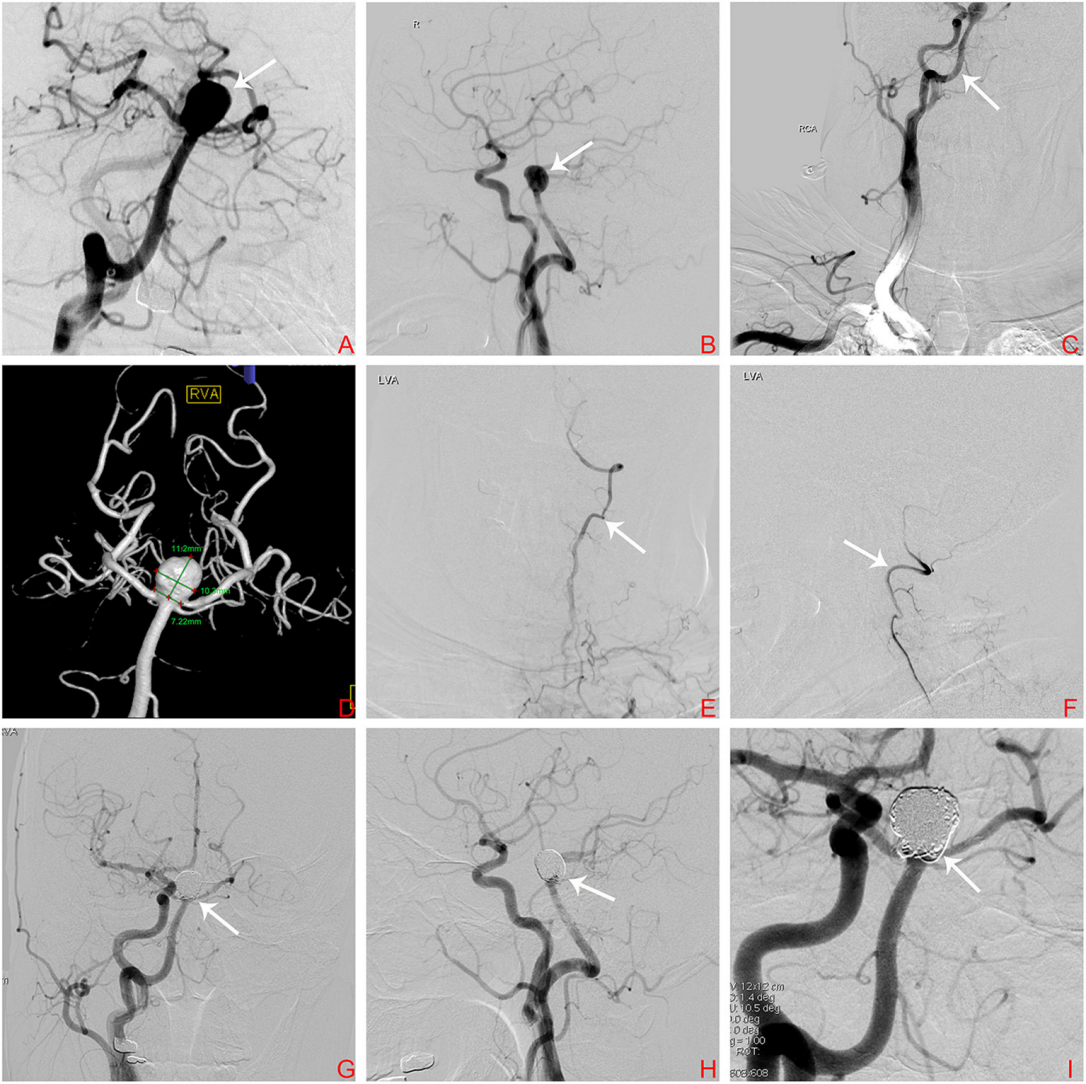
**(A)** Digital subtraction arteriogram (DSA) of the BA showing a basilar bifurcation aneurysm (white arrow); **(B)** DSA, lateral view of the right ICA showing a basilar bifurcation aneurysm (white arrow); **(C)** DSA lateral view of the right ICA showing the PPHA (white arrow) originating from the right ICA at the level of C1–C3; **(D)** Three-dimensional DSA demonstrating an unruptured basilar bifurcation aneurysm measuring 10.3 × 11.2 mm at the dome, with a 7.22-mm-wide neck (dome-to-neck ratio = 1.58); **(E,F)** DSA lateral views of the left VA showing hypoplasia of the left VA (white arrow); **(G–I)** Follow-up DSA demonstrating complete embolization of the aneurysm. The white arrow indicates no significant sign of recurrence. BA, basilar artery; ICA, internal carotid artery; PPHA, persistent primitive hypoglossal artery; VA, vertebral artery.

A 73-year-old woman suffered a fracture in an accidental fall. She had a medical history of hypertension and coronary heart disease. Her body mass index (BMI) was 32.5 kg/m^2^ and she weighed 78 kg; she had no family history of intracranial aneurysm. Magnetic resonance imaging (MRI) detected a basilar bifurcation aneurysm (Figure 2). During the postoperative follow-up after fracture repair, clinical examination findings, including neurological examination findings, were normal. Computed tomography angiography (CTA) and three-dimensional reconstruction was performed to evaluate this aneurysm. CTA revealed an unruptured wide-neck basilar bifurcation aneurysm and the presence of a PPHA originating from the right internal carotid artery, crossing the hypoglossal canal, and forming carotid–vertebrobasilar anastomoses (Figure 2). DSA demonstrated an unruptured basilar bifurcation aneurysm (Figures 3A–D) measuring 10.3 × 11.2 mm at the dome, with a 7.22-mm-wide neck (dome-to-neck ratio = 1.58). Given the morphological features of this complex wide-neck bifurcation aneurysm, and because the patient was older (>70 years) and recovering from the ankle fracture, after multidisciplinary evaluation (Neurosurgery Department, Interventional Neuroradiology Department, and Anesthesiology Department), we performed stent-assisted coil embolization in accordance with Chinese expert consensus, the patient's willingness, and her physical condition.

For such complex wide-neck bifurcation aneurysms, the Woven EndoBridge (WEB; Sequent Medical, Aliso Viejo, California) system is a good choice (11–14). Because the WEB is an intrasaccular flow diversion device, there is no need to administer dual-antiplatelet treatment before or after the procedure. However, the WEB device has not been approved by the National Medical Products Administration and is not available for clinical use in China.

In this patient, the left vertebral and posterior communicating arteries (PcomAs) were hypoplastic (Figures 3E,F); therefore, endovascular therapy via a PPHA approach was the only treatment option.

## Endovascular Treatment

**Figure 4:**
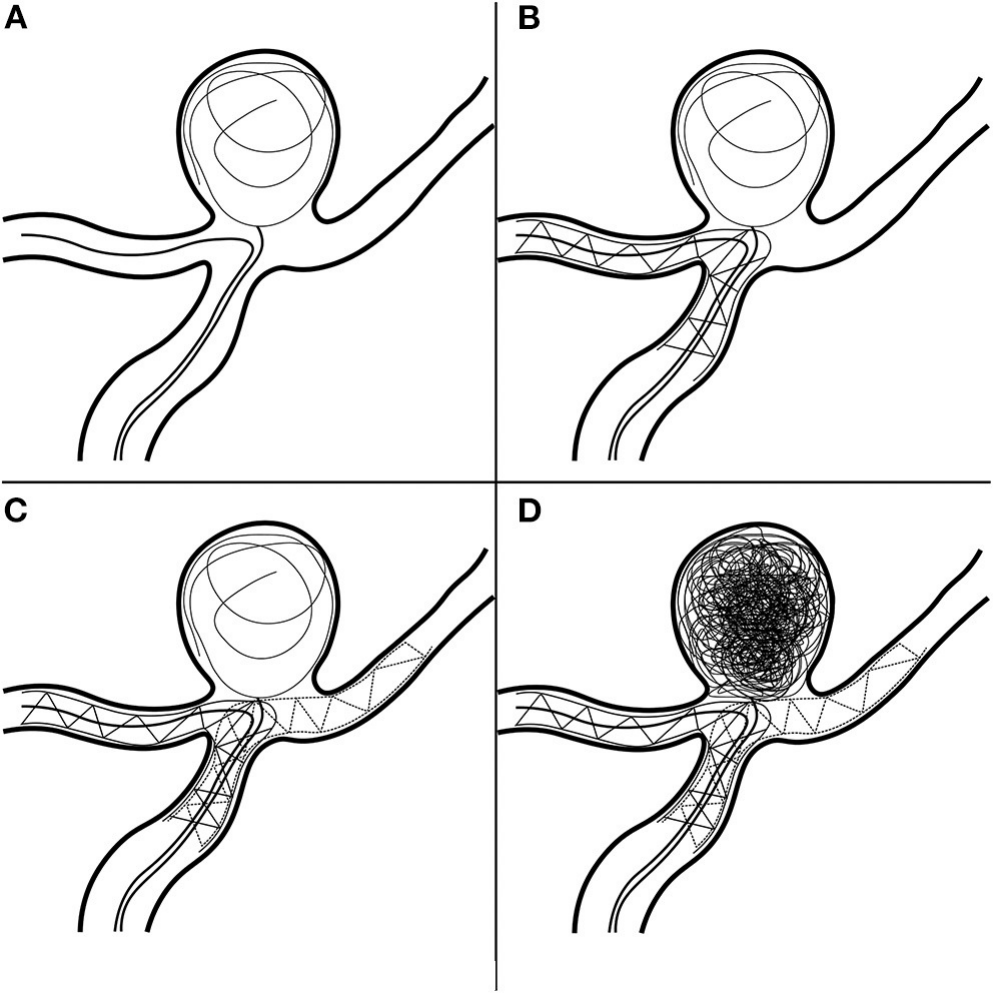
Procedures in the Y-stent-assisted coil embolization. **(A)** Wove a basket with a coil. **(B)** Deployment of first stent. **(C)** Deployment of second stent. **(D)** Complete occlusion of the aneurysm.

Dual-antiplatelet therapy (aspirin 100 mg/day, clopidogrel 75 mg/day) was administered 3 days prior to the operation. Under general anesthesia, a 6-Fr sheath was introduced into the right femoral artery; then, we guided a 6-Fr Cook shuttle (Cook Medical, Bloomington, IN, USA) to the C1 segment of the right ICA. Next, we introduced a 6-Fr Navien (Medtronic, Inc., Minneapolis, MN, USA) intracranial support catheter to the V2 segment of the right vertebral artery (VA). A Headway 17 microcatheter (MicroVention Inc., Tustin, CA, USA) was navigated over a Traxcess 14 guidewire (MicroVention Inc.) into the P1 segment of the right posterior cerebellar artery (PCA). Next, with the help of another Traxcess 14 guidewire, we navigated an Echelon 10 microcatheter (eV3 Neurovascular, Inc., Irvine, CA, USA) into the lumen of the aneurysm. To preclude coil detachment, we “wove a basket” with a coil (Figure 4A); the first coil measured 8 mm × 30 cm (QC-8-30-3D). Next, we removed the first Traxcess 14 guidewire, and a 4.5 × 22-mm Enterprise stent (Cordis Neurovascular, Miami, FL, USA) was deployed, extending from the right PCA to the BA trunk, not completely covering the aneurysm neck (Figure 4B). Subsequently, we navigated a second Headway 17 microcatheter and crossed the first stent into the left PCA with a second 4.5 × 28-mm Enterprise stent deployed into the right PCA, forming a Y-stent configuration (Figure 4C). With continuous coil placement, the aneurysm was finally coiled. Intraoperative DSA was obtained and demonstrated good stent positioning with very good wall apposition and complete occlusion of the aneurysm (Figure 4D). Throughout the procedure, 5,000 units of intravenous heparin were administered to obtain an activated coagulation time of 200–250 s. For better understanding, we illustrated the steps in the surgical procedure in a graphic flowchart (Figure 4). Postoperatively, the patient recovered well and was discharged to her home after 4 days of inpatient rehabilitation. The 6-month follow-up DSA demonstrated complete embolization of the aneurysm (Figures 3G–I). There was no significant sign of recurrence, and the patient's neurological examination was normal.

## Discussion

PPHA is a primitive communication between the ICA and the BA. Normally, the embryonic anastomotic vessels disappear after the formation of the posterior communicating artery (PcomA). When a segmental anastomotic branch does not disappear and persists to adulthood, it is called a persistent carotid–vertebrobasilar anastomosis. In 1889, the first report of PPHA was presented by Batujeff (15). Begg first demonstrated the angiography of PPHA in 1961 (16). In the following decades, a few cases of PPHA have been reported, with a very low incidence ranging from 0.027 to 0.26%.

The anatomic and angiographic definition of PPHA is according to the following diagnostic criteria: (1) the PPHA originates from the cervical segment of the ICA at the CI–C3 level; (2) the PPHA along with the twelfth cranial nerve enters the skull via traversing the hypoglossal canal, joining with the BA at the level of the occipital foramen; (3) the BA is filled only beyond the point where the artery joins it; and 4) the absence of a PcomA, and the ipsilateral VA is hypoplastic (17).

PPHA is usually asymptomatic, and most cases are encountered incidentally during angiography. Nonetheless, the most common clinical characteristic of PPHA is aneurysm (10). Although whether PPHA predisposes patients to an increased risk of intracranial aneurysms remains controversial, and it is noteworthy that hemodynamic changes and abnormalities in the vessel wall strongly correlate with aneurysm formation. In our PPHA case, the absence of a right VA and bilateral PcomAs and hypoplasia of the left VA indicated that endovascular therapy via PPHA approach was the only treatment option.

Our literature review identified several reports of the treatment of PPHA accompanied by a basilar bifurcation aneurysm (Table 1) (3, 5–7, 18–21). The reported techniques were surgical clipping and coil embolization. Except for one patient whose condition progressed rapidly and who did not receive treatment and died 39 days after aneurysm onset, all patients achieved good outcomes after appropriate therapy. Overall, these results were encouraging. It must be noted that complex wide-neck bifurcation aneurysms in the posterior circulation pose a substantial challenge with both open microsurgical and endovascular treatment. With evolutions in treatment and developments in endovascular techniques, deploying Y-stents significantly improves patients' prognosis by acting as a scaffold to prevent coil prolapse, preserving the parent artery, promoting thrombosis in the aneurysm lumen, and preventing recanalization (22–25). Y-stent-assisted coil embolization is a suitable candidate for complex wide-neck bifurcation aneurysms.

**Table 1:** Literature review of the treatment of basilar bifurcation aneurysm accompanied by PPHA.

**Series**	**Year**	**Age/Sex**	**Side of PPHA**	**Symptoms**	**Treatment**	**Clinical results**
Anderson, M.	1976	29/F	Left	SAH	Ventriculo-cardiostomy	Dead
Kodama T. et al.	1976	—	Right	—	Clipping	Good
Kobayashi H. et al.	1984	46/M	Right	SAH	Clipping	Good
Yeh H. et al.	1987	61/F	Left	SAH	Clipping	Good
Harada K. et al.	1994	73/F	Right	SAH	Clipping	Lost to follow-up
Sakai K. et al.	1998	71/M	Left	SAH	Clipping	Good
Sakai, K. et al.	1998	43/F	Right	SAH	Clipping	Good
Bapuraj J. R. et al.	2007	60/F	Right	SAH	Coil embolization	Good
Wang M. et al.	2017	34/F	Right	SAH	Clipping	Good
This study	2016	73/F	Right	Asymptomatic	Y-stent	Good

Surgical clipping and endovascular treatment remain the predominant modality in the management of intracranial aneurysms (26, 27). Management of posterior circulation aneurysms is still a formidable challenge, especially aneurysms of the bifurcation of the BA, because these aneurysms are adjacent to the brainstem and the cranial nerves, and blood supply to the brainstem derives from its pontine perforators. Chow et al. demonstrated the first successful application of Y-stent-assisted coil embolization in 2004, and this technique was proven safe and effective in several subsequent studies.

To our knowledge, ours is the first report of successful embolization of a basilar bifurcation aneurysm associated with a persistent PPHA treated with Y-stent-assisted coiling.

## Conclusion

In this case, both clinical outcomes and short-term follow-up findings were encouraging. Y-stent-assisted coil embolization for an unruptured basilar bifurcation aneurysm via PPHA is feasible. However, long-term observation will be required for this single case.

## Data Availability Statement

The original contributions presented in the study are included in the article/supplementary material, further inquiries can be directed to the corresponding author/s.

## Ethics Statement

The studies involving human participants were reviewed and approved by Beijing Tiantan ethics committee. The patients/participants provided their written informed consent to participate in this study. Written informed consent was obtained from the individual(s) for the publication of any potentially identifiable images or data included in this article.

## Author Contributions

LZ and XC performed the manuscript writing. LJ acquired the data. LD and JW analyzed and interpreted the data. LZ edited the figure of the article. ML and PL conceived and designed the research. All authors contributed to the article and approved the submitted version.

## Conflict of Interest

The authors declare that the research was conducted in the absence of any commercial or financial relationships that could be construed as a potential conflict of interest.
